# Supplementary material for: Cyclic pentapeptide cRGDfK enhances the inhibitory effect of sunitinib on TGF-β1-induced epithelial-to-mesenchymal transition in human non-small cell lung cancer cells
Source: PLoS One. 2020 Aug 18;15(8):e0232917. doi: 10.1371/journal.pone.0232917 (PMC7433881; doi:10.1371/journal.pone.0232917)
Supplement: S1 Fig — The detected bands were quantified based on the ImageJ software, and the relative ratio between each sample and loading controls was presented in the figures. (DOCX) [file pone.0232917.s001.docx]

**
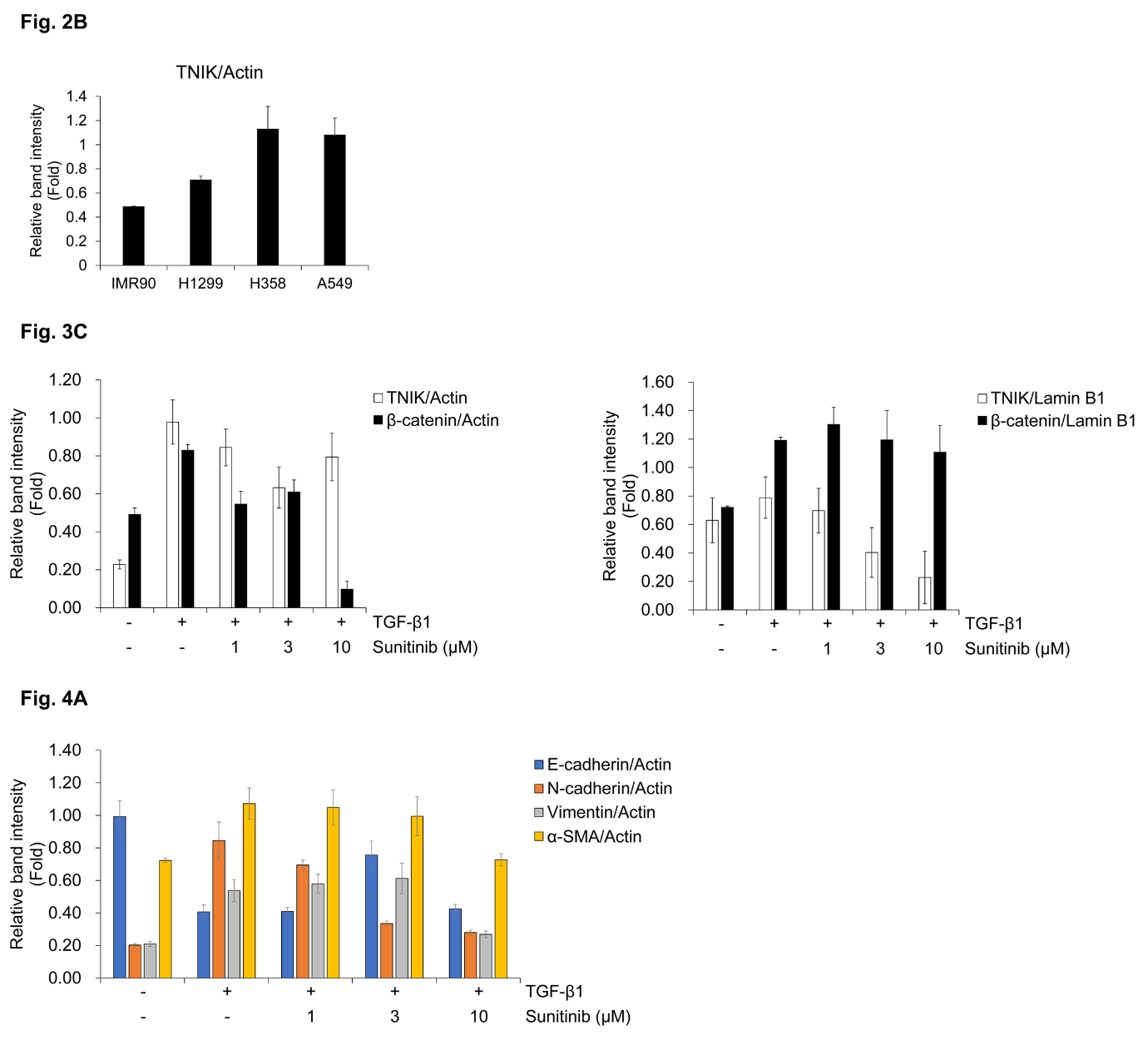
**continued

**
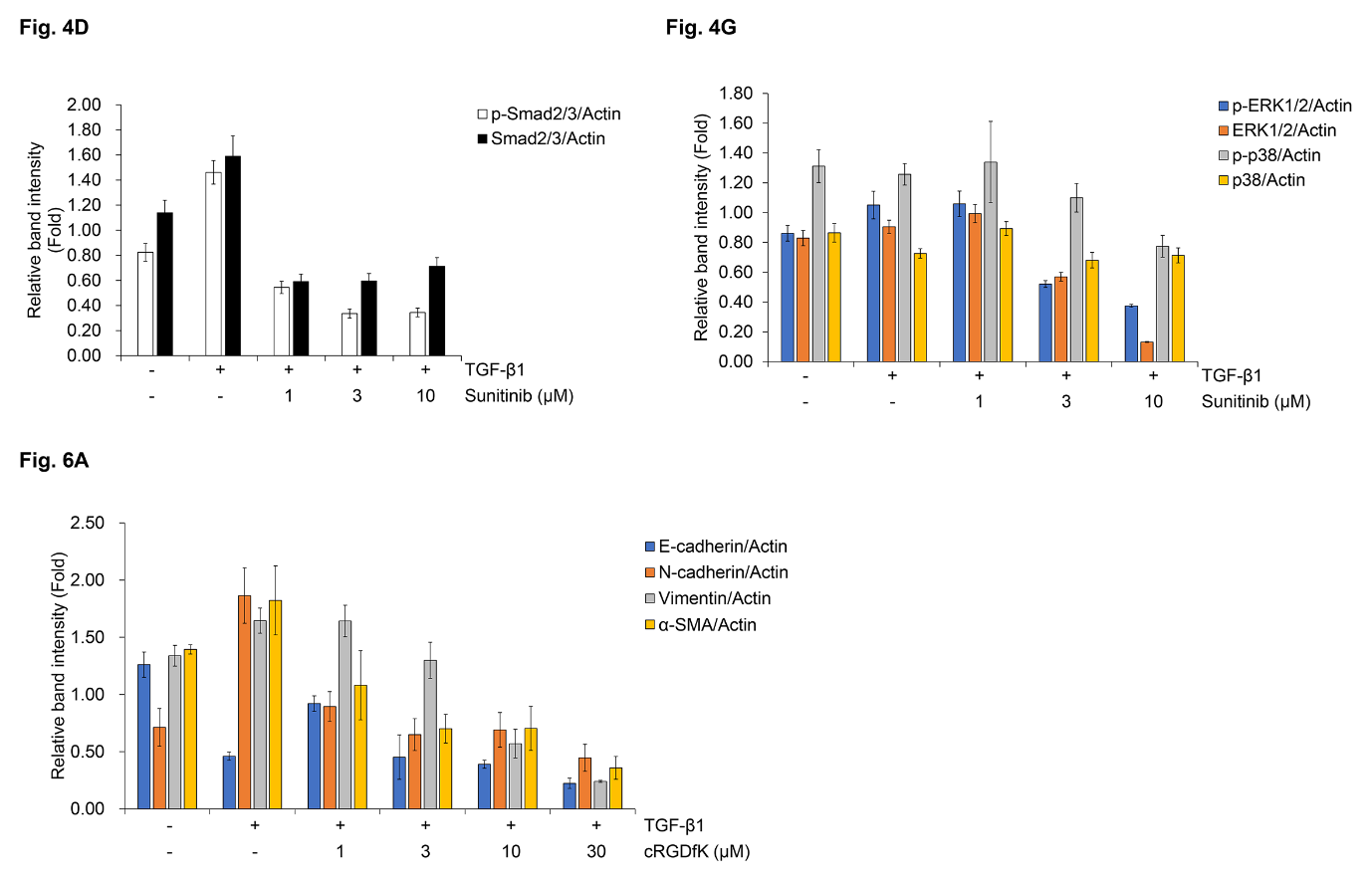
**

**Figure S1.** The average band intensities of the independent three western blot results in Fig. 2B, 3C, 4A, 4D, 4G and 6A. The detected bands were quantified based on the ImageJ software, and the relative ratio between each sample and loading controls was presented in the figures.
