# Supplementary material for: Cyclic pentapeptide cRGDfK enhances the inhibitory effect of sunitinib on TGF-β1-induced epithelial-to-mesenchymal transition in human non-small cell lung cancer cells
Source: PLoS One. 2020 Aug 18;15(8):e0232917. doi: 10.1371/journal.pone.0232917 (PMC7433881; doi:10.1371/journal.pone.0232917)
Supplement: S2 Fig — A549 (A) and H358 (B) cells were transfected with non-targeting control siRNA (siCont) or TNIK siRNA (siTNIK). Transfected cells were treated with sunitinib for 24 h. Cell viability was measured by CCK-8 assay. Experiments were performed in triplicate. Data represent the mean ± SD of raw results. * p < 0.05 and ** p < 0.001 (vs. control). (DOCX) [file pone.0232917.s002.docx]

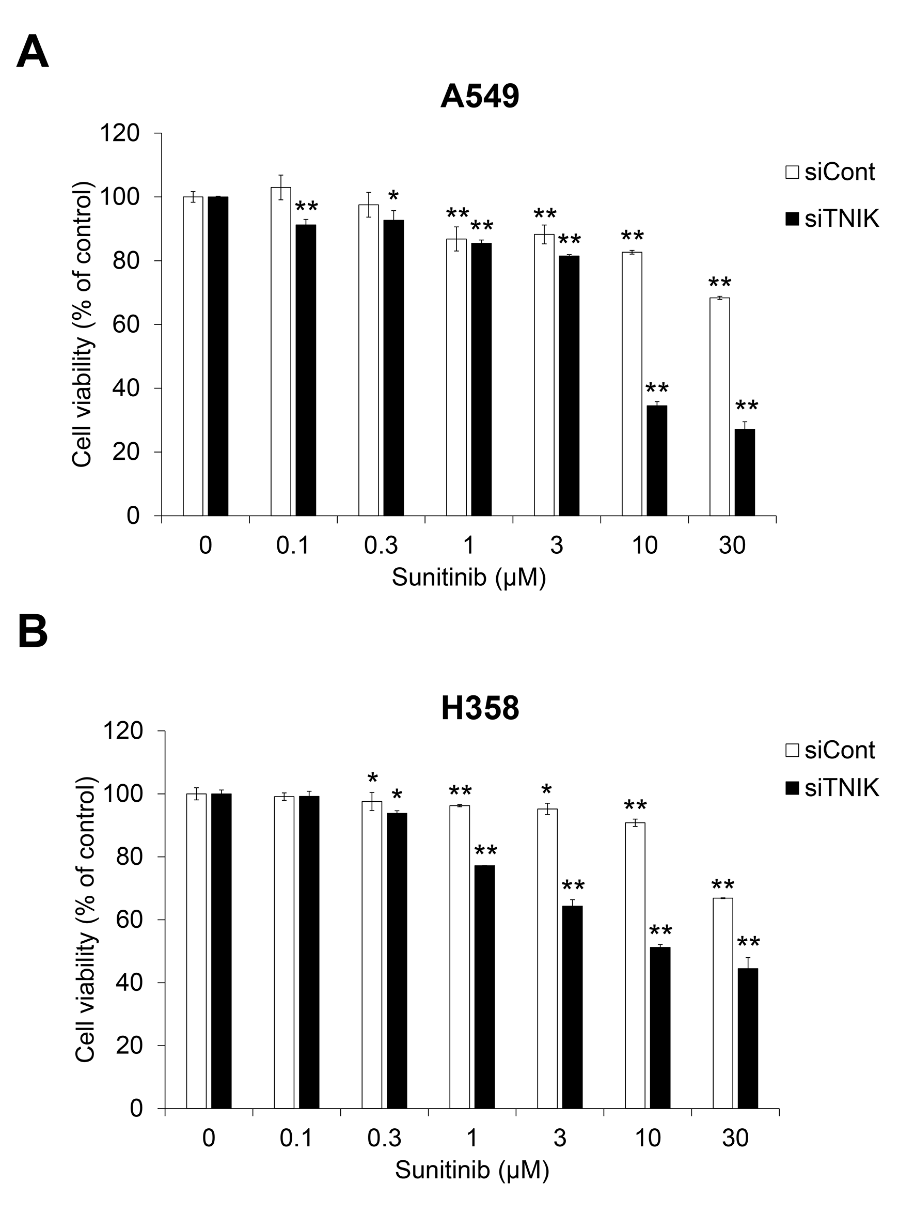


**Figure S2. The effect of TNIK-silencing on the sensitivity to sunitinib.** A549 (A) and H358 (B) cells were transfected with non-targeting control siRNA (siCont) or TNIK siRNA (siTNIK). Transfected cells were treated with sunitinib for 24 h. Cell viability was measured by CCK-8 assay. Experiments were performed in triplicate. Data represent the mean ± SD of raw results. * *p* < 0.05 and ** *p* < 0.001 (vs. control).
