# Supplementary material for: Cyclic pentapeptide cRGDfK enhances the inhibitory effect of sunitinib on TGF-β1-induced epithelial-to-mesenchymal transition in human non-small cell lung cancer cells
Source: PLoS One. 2020 Aug 18;15(8):e0232917. doi: 10.1371/journal.pone.0232917 (PMC7433881; doi:10.1371/journal.pone.0232917)
Supplement: S3 Fig — A549 cells were transfected with non-targeting control siRNA or TNIK siRNA. Transfected cells were treated with TGF-β1 (5 ng/mL) for 48 h. The mRNA expression was measured by qRT-PCR analysis. Experiments were performed in triplicate. Data represent mean ± SD. (DOCX) [file pone.0232917.s003.docx]

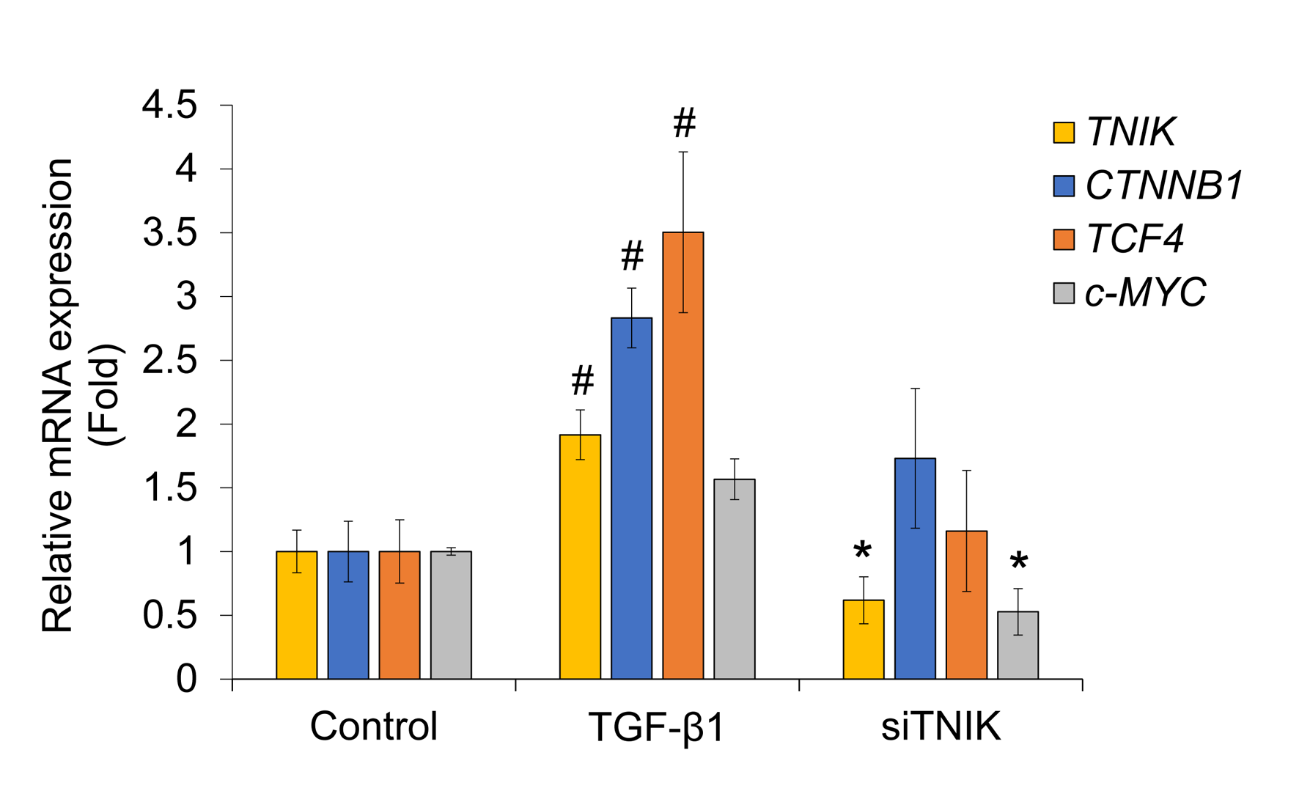


**Figure S3. The mRNA expression of TNIK and Wnt target genes in A549 cells.** A549 cells were transfected with non-targeting control siRNA or TNIK siRNA. Transfected cells were treated with TGF-β1 (5 ng/mL) for 48 h. The mRNA expression was measured by qRT-PCR analysis. Experiments were performed in triplicate. Data represent mean ± SD of raw dataset.
