# Supplementary material for: Cyclic pentapeptide cRGDfK enhances the inhibitory effect of sunitinib on TGF-β1-induced epithelial-to-mesenchymal transition in human non-small cell lung cancer cells
Source: PLoS One. 2020 Aug 18;15(8):e0232917. doi: 10.1371/journal.pone.0232917 (PMC7433881; doi:10.1371/journal.pone.0232917)
Supplement: S5 Fig — The protected linear pentapeptide (1) bound to the resin was synthesized using the Fmoc solid phase peptide synthesis (SPPS) method. The linear peptide (2) was cleaved from the resin without affecting other protecting groups by using acetic acid/TFE/CH2Cl2 (1:1:3 ratio) solution. Finally, cyclic pentapeptide c(RGDfK) (4) was obtained by head-to tail cyclization under T3P, TEA, DAMP and elimination of the protecting group. (DOCX) [file pone.0232917.s005.docx]

**Figure S5. Synthetic scheme for cyclic pentapeptide, c(RGDfK) (4).** The protected linear pentapeptide (**1**) bound to the resin was synthesized using the Fmoc solid phase peptide synthesis (SPPS) method. The linear peptide (**2**) was cleaved from the resin without affecting other protecting groups by using acetic acid/TFE/CH_2_Cl_2_ (1:1:3 ratio) solution. Finally, cyclic pentapeptide c(RGDfK) (**4**) was obtained by head-to tail cyclization under T3P, TEA, DAMP and elimination of the protecting group.
