# Supplementary material for: Cyclic pentapeptide cRGDfK enhances the inhibitory effect of sunitinib on TGF-β1-induced epithelial-to-mesenchymal transition in human non-small cell lung cancer cells
Source: PLoS One. 2020 Aug 18;15(8):e0232917. doi: 10.1371/journal.pone.0232917 (PMC7433881; doi:10.1371/journal.pone.0232917)
Supplement: S6 Fig — Serum-deprived A549 cells were treated with TGF-β1 (5 ng/mL) or its combination with cRGDfK for 72 h. Actin was used as a loading control. (DOCX) [file pone.0232917.s006.docx]

**
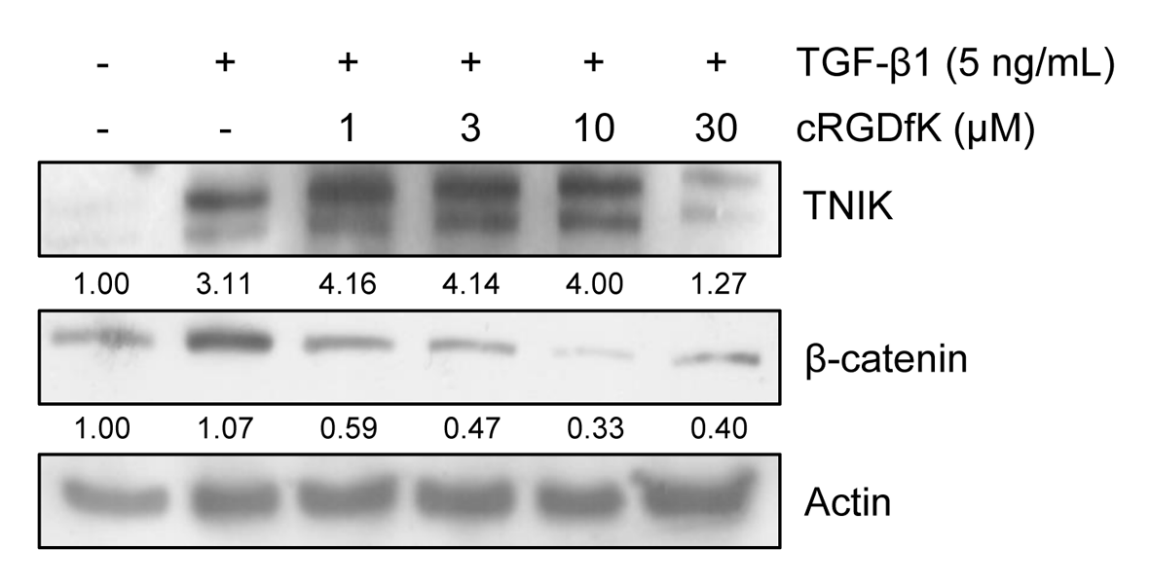
**

**Figure S6.** **Western blot analysis of cytosolic TNIK and β-catenin expression.** Serum-deprived A549 cells were treated with TGF-β1 (5 ng/mL) or its combination with cRGDfK for 72 h. Actin was used as a loading control.
