# Supplementary material for: Cyclic pentapeptide cRGDfK enhances the inhibitory effect of sunitinib on TGF-β1-induced epithelial-to-mesenchymal transition in human non-small cell lung cancer cells
Source: PLoS One. 2020 Aug 18;15(8):e0232917. doi: 10.1371/journal.pone.0232917 (PMC7433881; doi:10.1371/journal.pone.0232917)
Supplement: S8 Fig — H358 (A) and H1299 (B) cells were treated with sunitinib and cRGDfK for 24 h. After incubation, cell viability was measured by CCK-8 assay. Experiments were performed in triplicate. Data represent mean ± SD. * p < 0.05 and ** p < 0.001 (vs. control). (DOCX) [file pone.0232917.s008.docx]

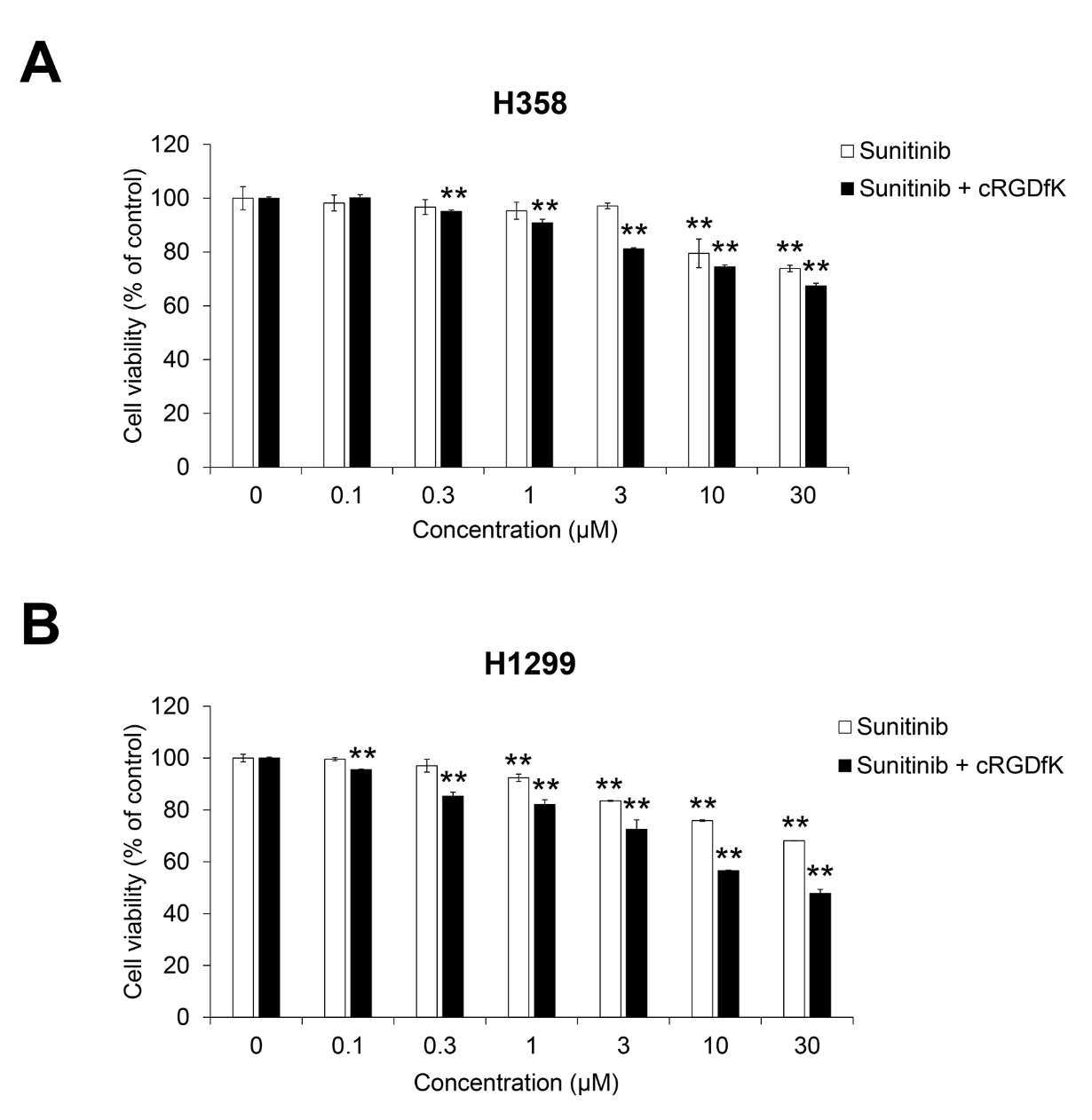


**Figure S8. Combination effect of sunitinib with cRGDfK on cell viability in NSCLC H358 and H1299 cells.** H358 (A) and H1299 (B) cells were treated with sunitinib and cRGDfK for 24 h. After incubation, cell viability was measured by CCK-8 assay. Experiments were performed in triplicate. Data represent mean ± SD. * *p* < 0.05 and ** *p* < 0.001 (vs. control).
