# Supplementary material for: Cyclic pentapeptide cRGDfK enhances the inhibitory effect of sunitinib on TGF-β1-induced epithelial-to-mesenchymal transition in human non-small cell lung cancer cells
Source: PLoS One. 2020 Aug 18;15(8):e0232917. doi: 10.1371/journal.pone.0232917 (PMC7433881; doi:10.1371/journal.pone.0232917)
Supplement: S1 Table — (PDF) [file pone.0232917.s009.pdf]

S1 Table. Primer sequences used in this study.

Target gene	Forward (5' – 3')	Reverse (5' – 3')
TCF4	GAGCAGCAAGTCCGAGAAAG	ATGCTGAAACCTCTTGCGTC
CTNNB1	CCATCTTCCAGGAGCGAGAT	CAGTGATGGCATGGACTGTG
TNIK	GCTATTGAGATCCGGTCAGT	CAGGCTGCAACATTGAAAGA
c-MYC	AATGAAAAGGCCCCCAAGGTAGTTATCC	GTCGTTTCCGCAACAAGTCCTCTTC
TWIST1	ATTCAGACCCTCAAGCTGGC	GAGAGGGGAGGAAATCGAG
SNAI1	ACCTCCAGACCCACTCAGAT	GCAGAGGACACAGAACCAGA
CDH1	TCCGAAGCTGCTAGTCTGAG	CTCAAGGGAAGGGAGCTGAA
CDH2	CCCACAGCTCCACCATATGA	TTCAGTCATCACCTCCACCA
VIM	CGCCAACTACATCGACAAGG	GGCTTTGTCGTTGGTTAGCT
ITGAV	GCCGTGGATTTCTTCGTG	GAGGACCTGCCCTCCTTC
ITGB3	CGCTAAATTTGAGGAAGAACG	GAAGGTAGACGTGGCCTCTTT
GAPDH	GAGTCAACGGATTTGGTCGT	GATCTCGCTCCTGGAAGATG
