# Supplementary material for: Cyclic pentapeptide cRGDfK enhances the inhibitory effect of sunitinib on TGF-β1-induced epithelial-to-mesenchymal transition in human non-small cell lung cancer cells
Source: PLoS One. 2020 Aug 18;15(8):e0232917. doi: 10.1371/journal.pone.0232917 (PMC7433881; doi:10.1371/journal.pone.0232917)
Supplement: S2 Table — (PDF) [file pone.0232917.s010.pdf]

S2 Table. Combination index (CI) values for the two-drug combination against cell viability of H358 and H1299 cells.

Cell line	Sunitinib (μM)	cRGDfK (μM)	CI value
H358	0.3	0.3	0.7788
	1	1	0.6901
	3	3	0.4683
	10	10	0.6667
	30	30	0.6243
H1299	0.3	0.3	0.3180
	1	1	0.2130
	3	3	0.1646
	10	10	0.1051
	30	30	0.1306
