# Supplementary material for: Cyclic pentapeptide cRGDfK enhances the inhibitory effect of sunitinib on TGF-β1-induced epithelial-to-mesenchymal transition in human non-small cell lung cancer cells
Source: PLoS One. 2020 Aug 18;15(8):e0232917. doi: 10.1371/journal.pone.0232917 (PMC7433881; doi:10.1371/journal.pone.0232917)

Figure 2B

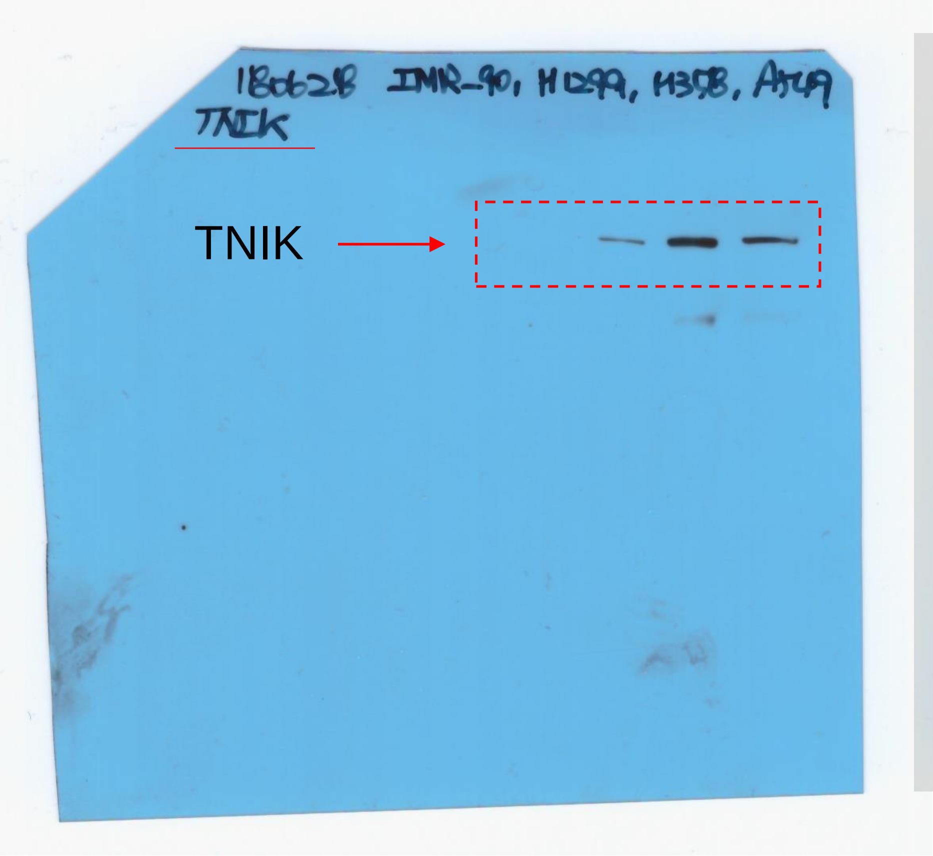
IMR-90, H1299, H358, A549
TNIK
TNIK

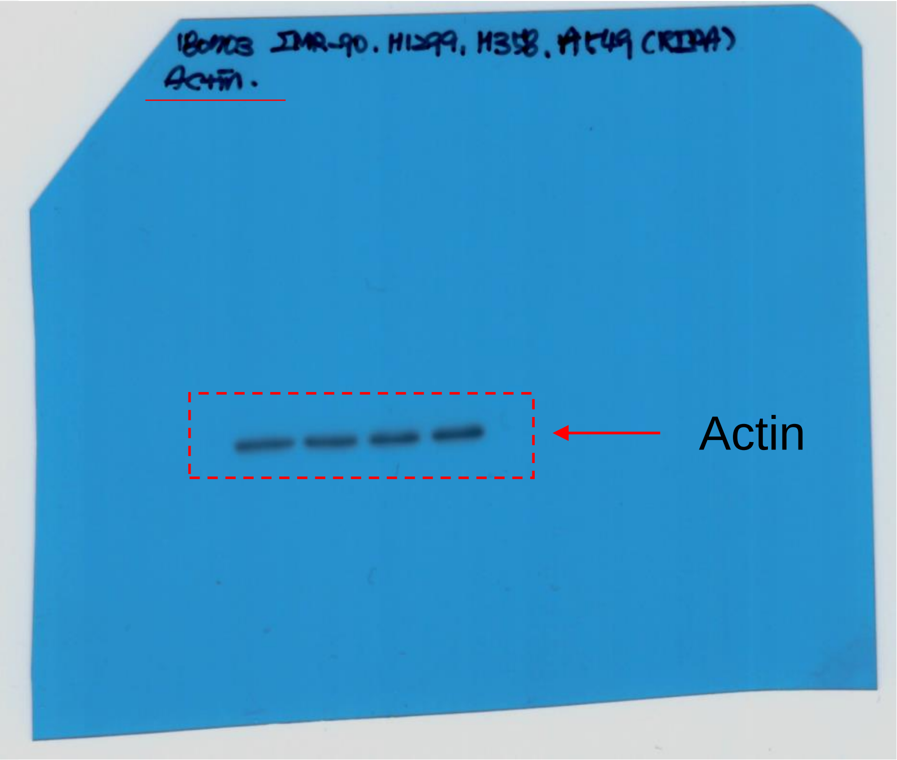
IMR-90. H1299. H358. A549 (RIPA)
Actin.
Actin

Figure 3C

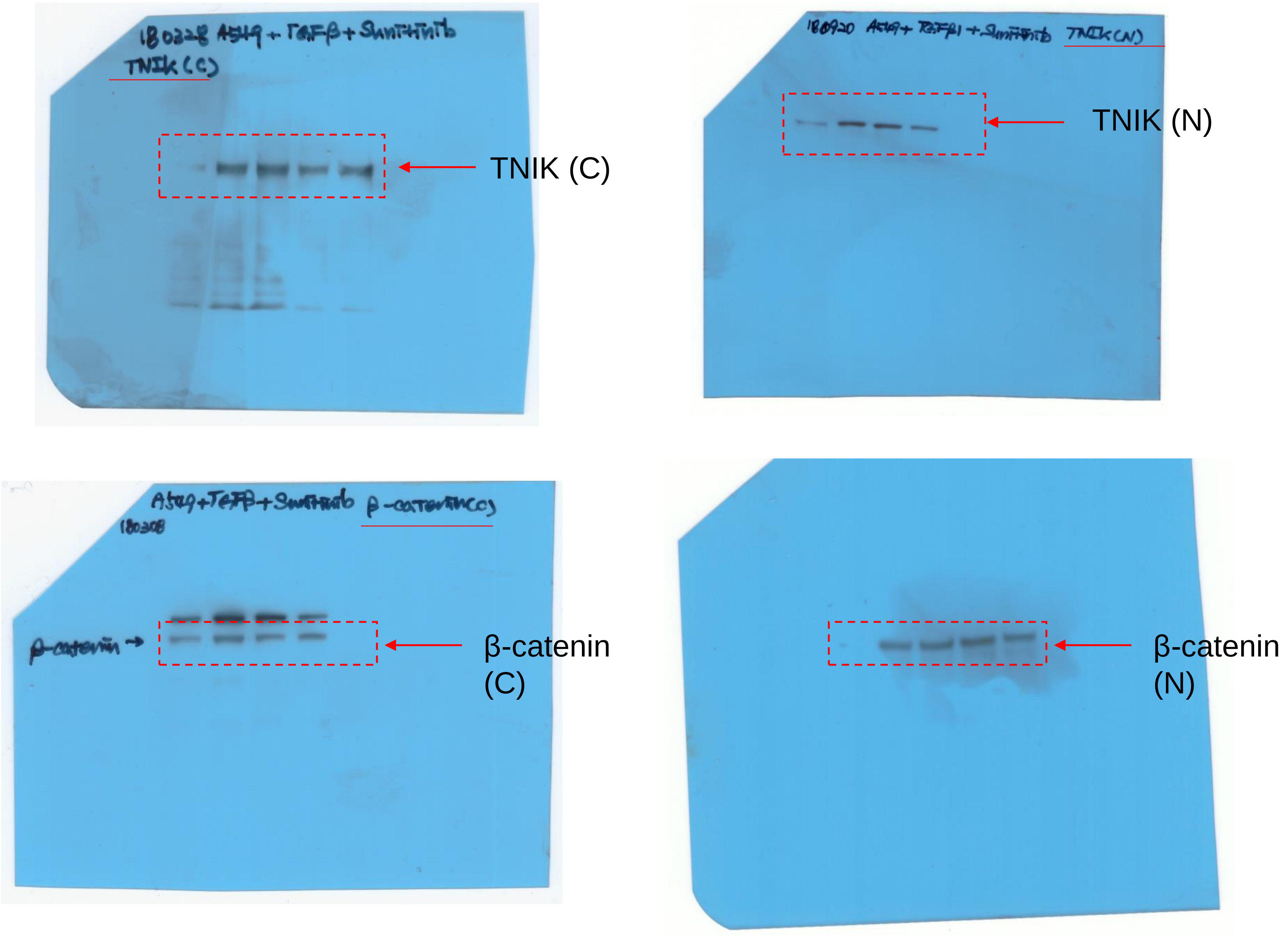

Figure 3C

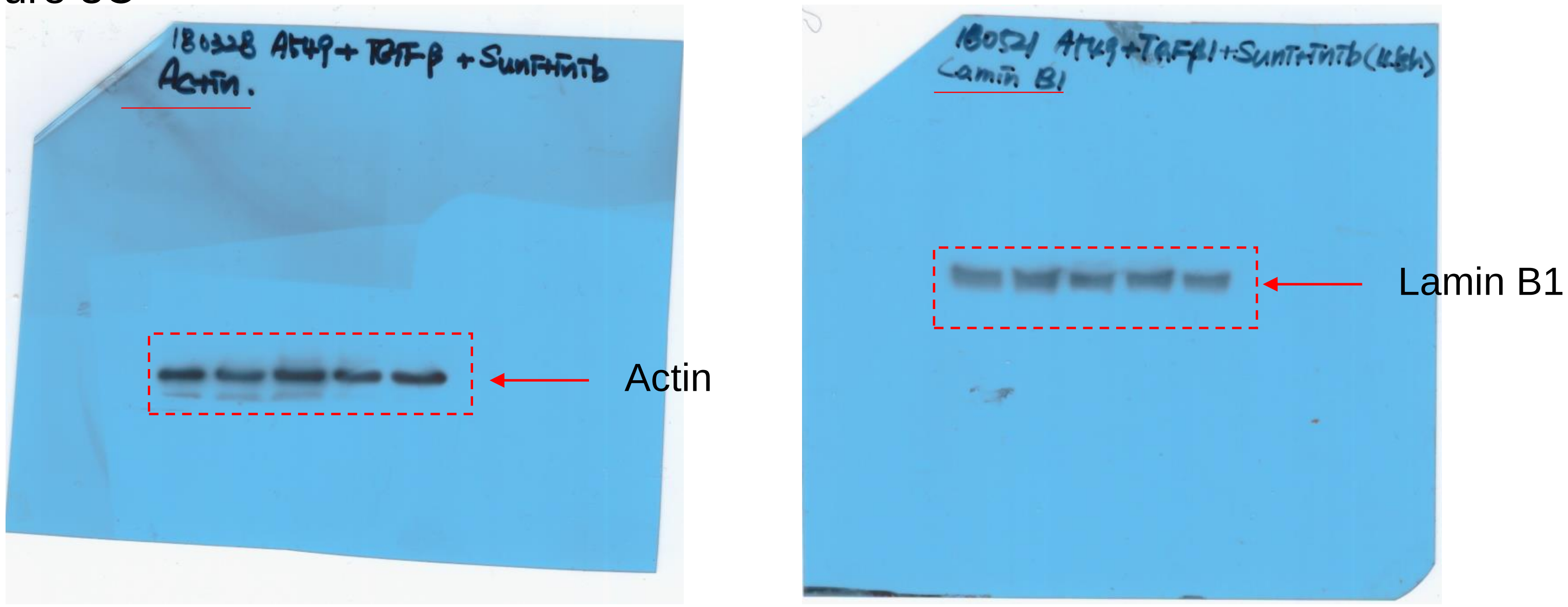

Figure 4A

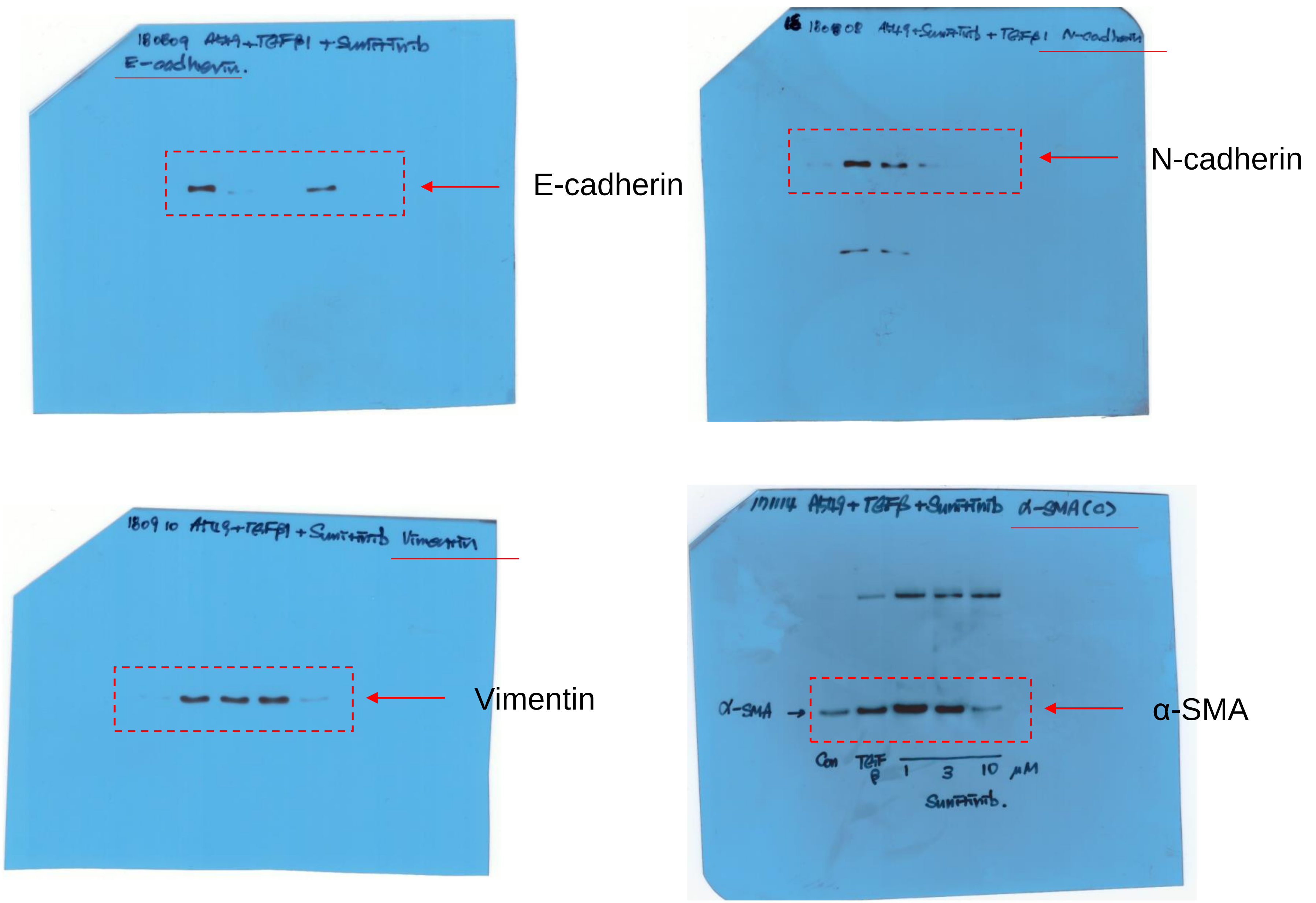

Figure 4A

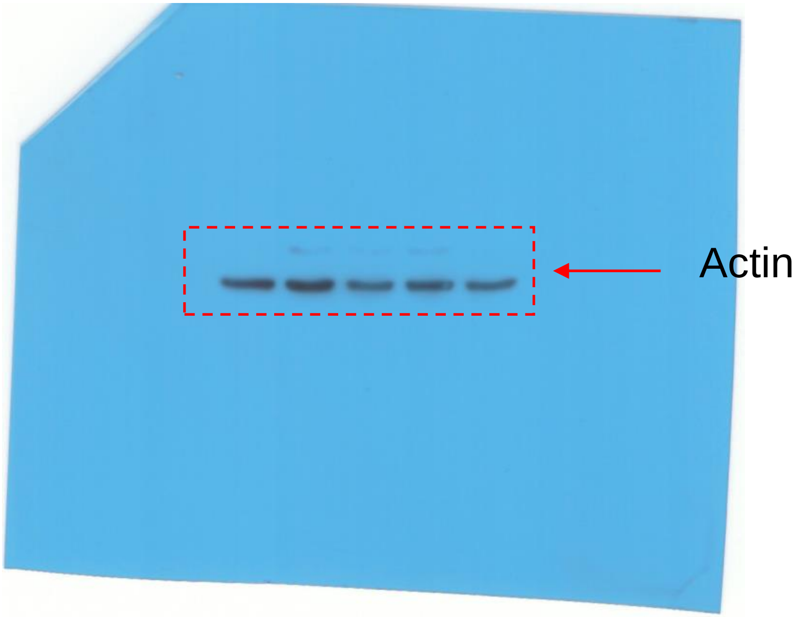

Figure 4D

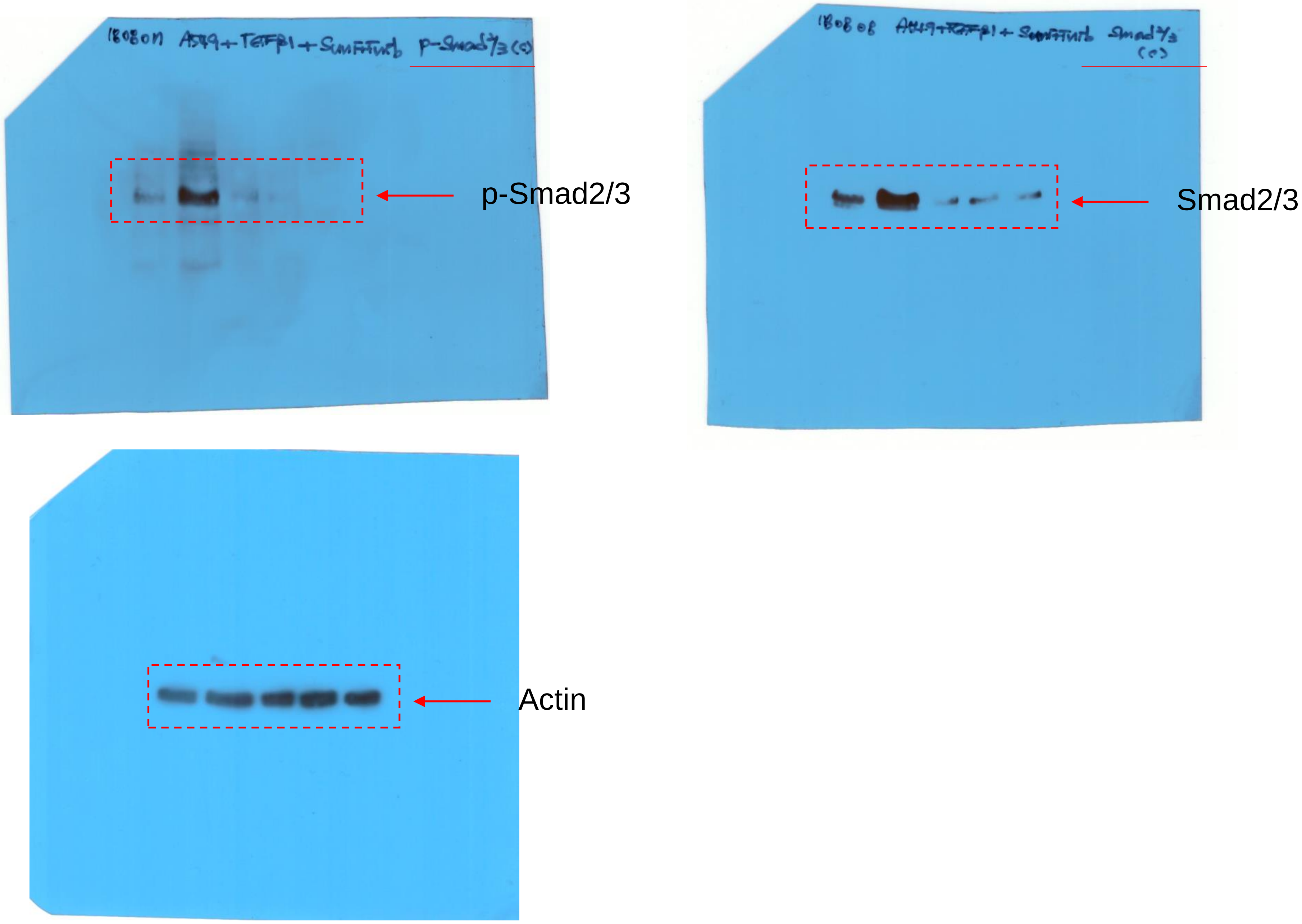

Figure 4G

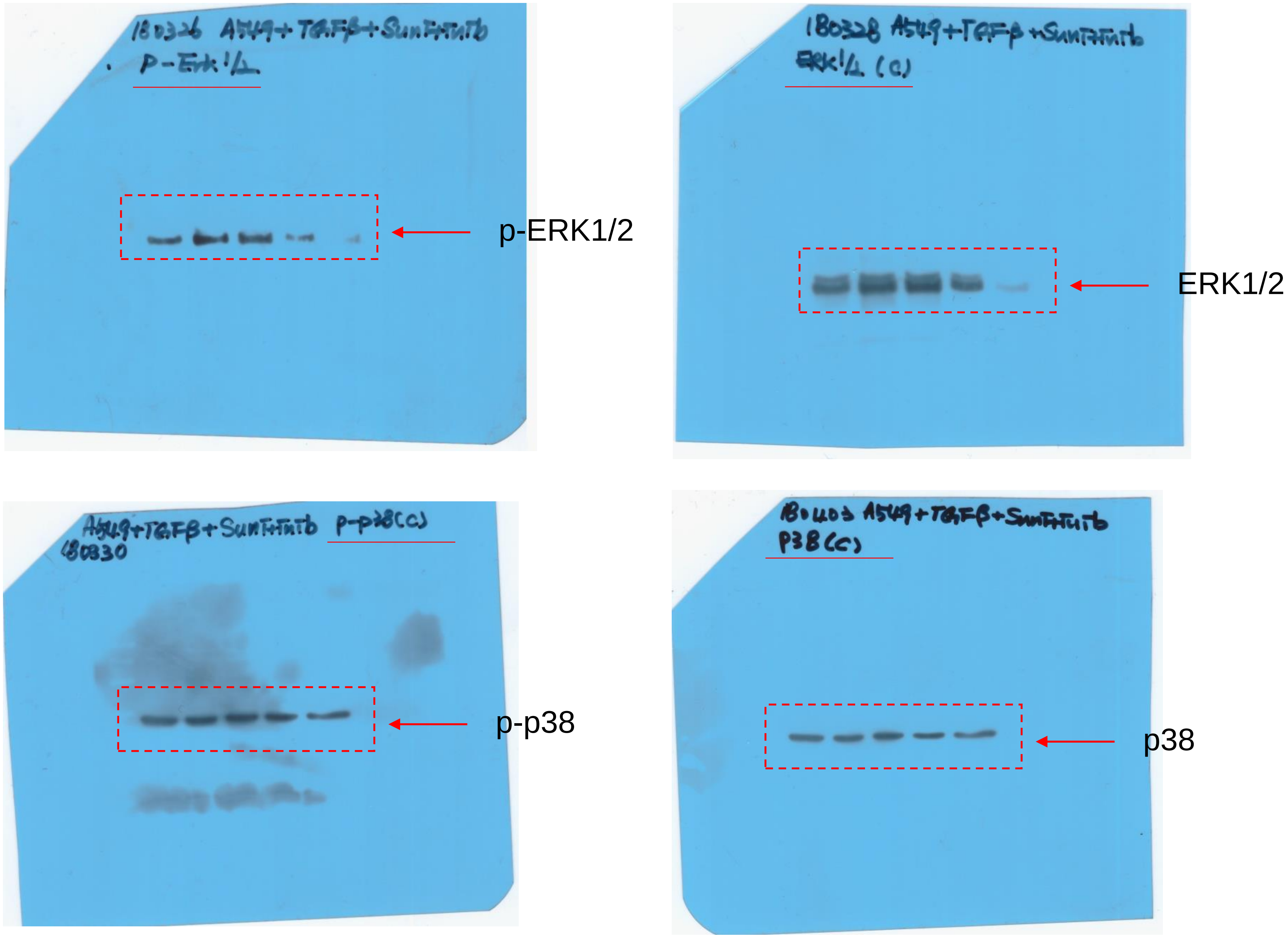

Figure 4G

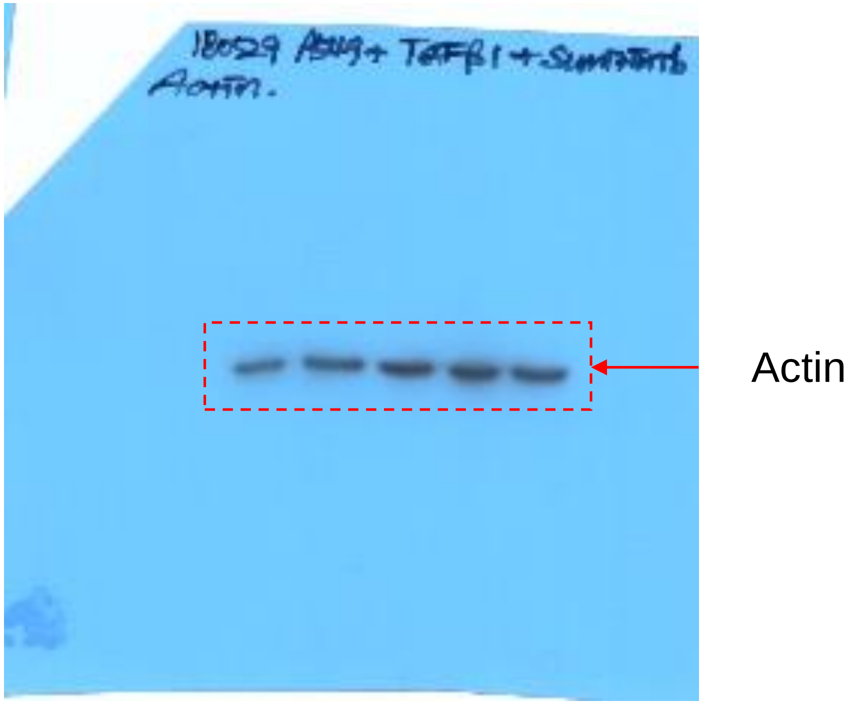

Figure 6A

1. control 2. TGF-β1 (5ng/ml) 3. TGF-β1+ cRGDfk 1uM 4. TGF-β1+ cRGDfk 3uM
5. TGF-β1+ cRGDfk 10uM 6. TGF-β1 + cRGDfk 30uM

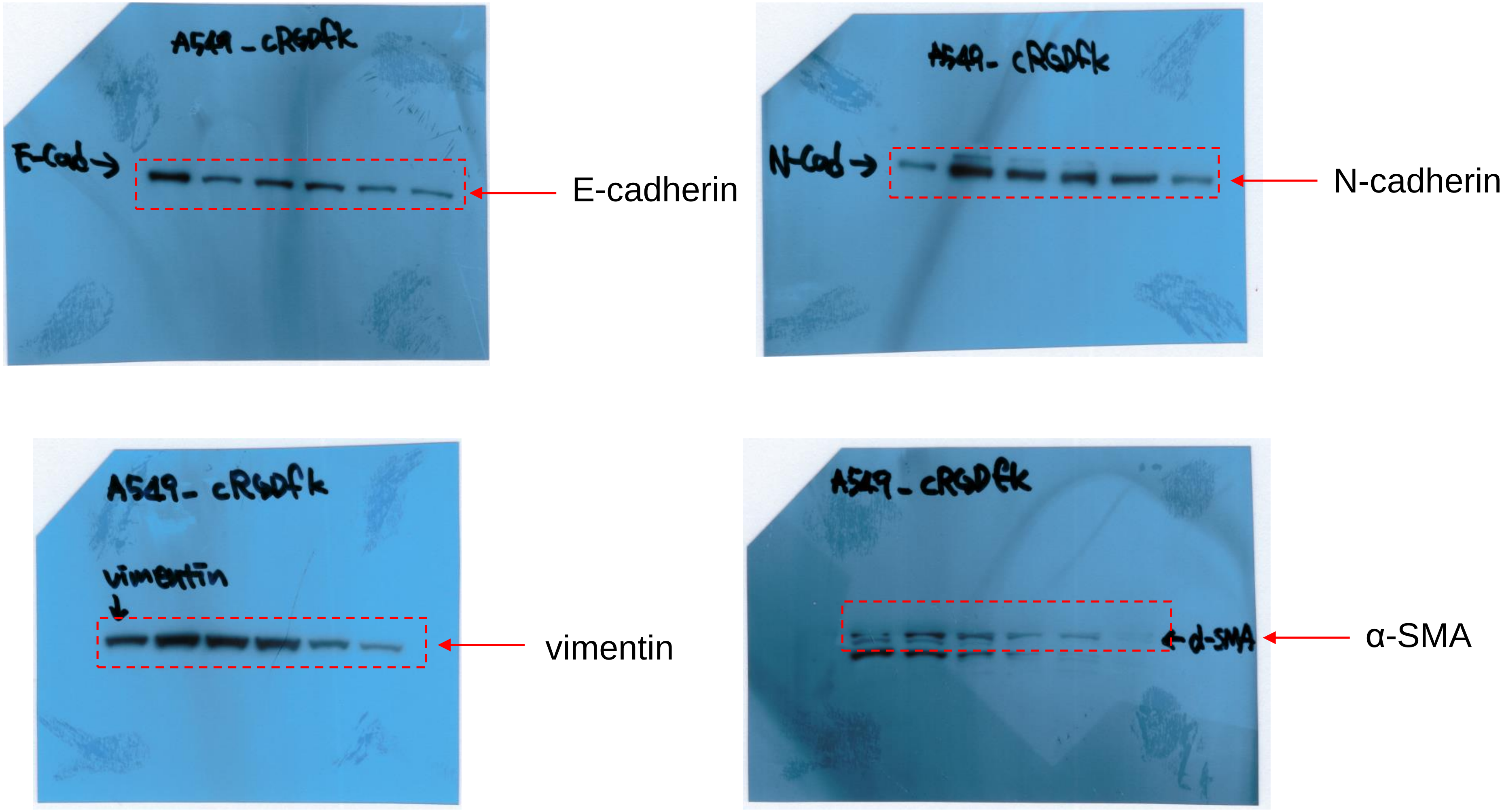

Figure 6A

1. control 2. TGF-β1 (5ng/ml) 3. TGF-β1+ cRGDfk 1uM 4. TGF-β1+ cRGDfk 3uM
5. TGF-β1+ cRGDfk 10uM 6. TGF-β1 + cRGDfk 30uM

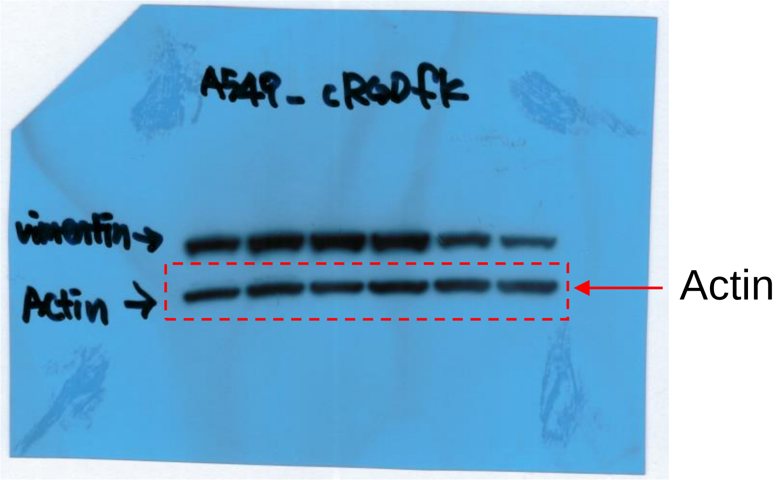

Figure 7B

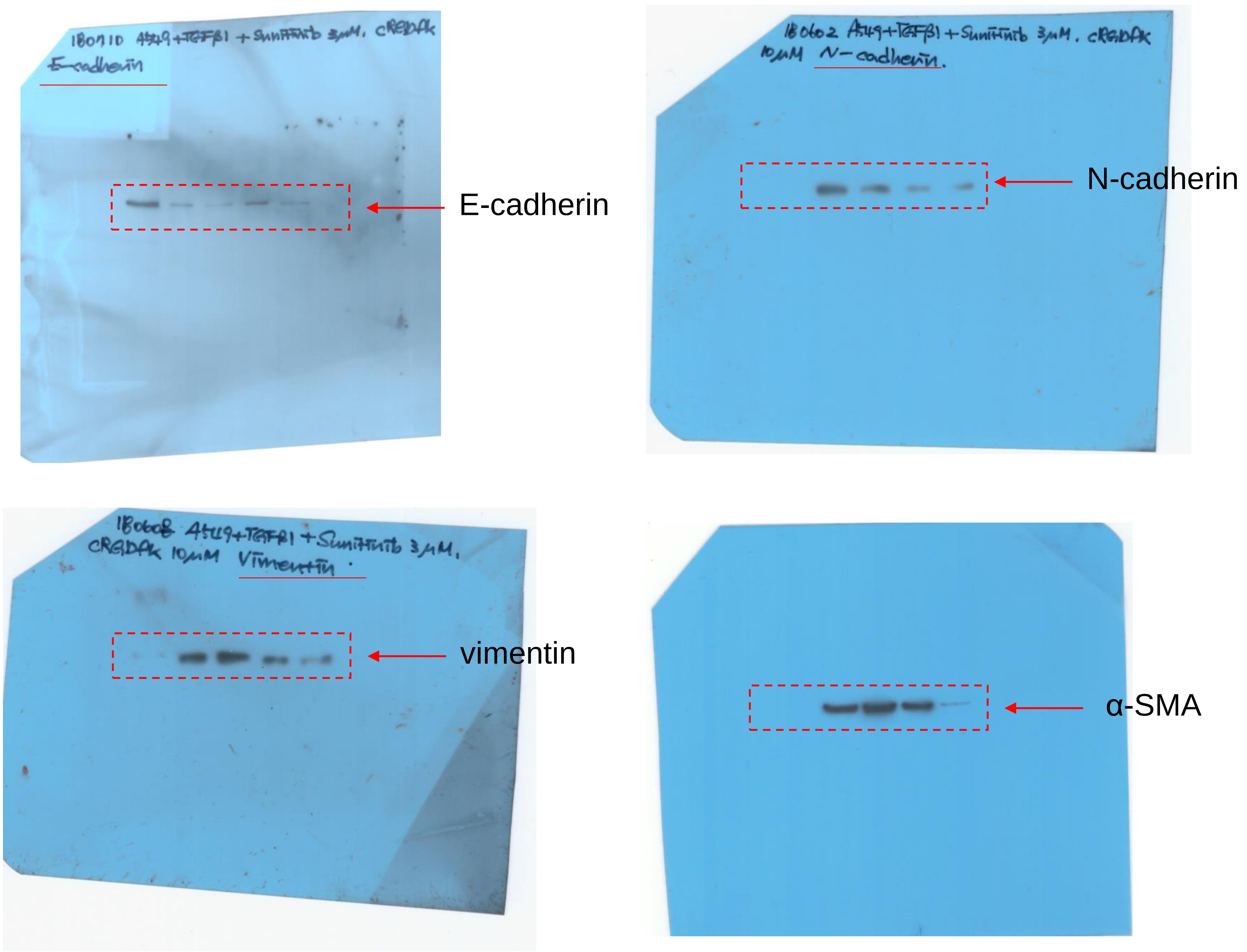

Figure 7B

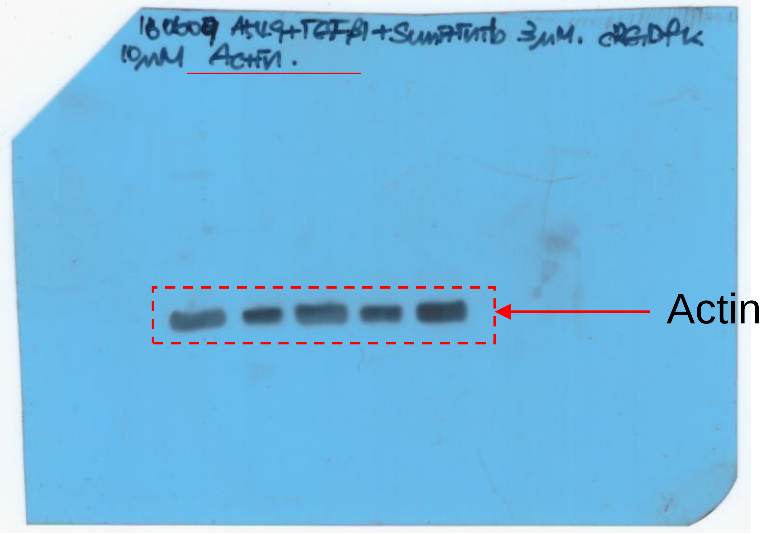

Supplement: S1 Raw images — (PDF) [file pone.0232917.s011.pdf]
